# Problems and challenges in the development and validation of human cell-based assays to determine nanoparticle-induced immunomodulatory effects

**DOI:** 10.1186/1743-8977-8-8

**Published:** 2011-02-09

**Authors:** Gertie J Oostingh, Eudald Casals, Paola Italiani, Renato Colognato, René Stritzinger, Jessica Ponti, Tobias Pfaller, Yvonne Kohl, Daniëlla Ooms, Flavia Favilli, Hilde Leppens, Davide Lucchesi, François Rossi, Inge Nelissen, Hagen Thielecke, Victor F Puntes, Albert Duschl, Diana Boraschi

**Affiliations:** 1 Department of Molecular Biology, University of Salzburg, 5020 Salzburg, Austria; 2 Institut Català de Nanotecnologia (ICN), 08193 Bellaterra, Spain; 3 Laboratory of Innate Immunity and Cytokines, Institute of Biomedical Technologies, National Research Council (CNR), 56124 Pisa, Italy; 4 European Commission, Joint Research Centre (JRC), Institute for Health and Consumer Protection, Nanobiosciences Unit, 21027 Ispra, VA, Italy; 5 Fraunhofer Institute for Biomedical Engineering, Department of Biohybrid Systems, 66386 St. Ingbert, Germany; 6 Centre for Advanced R&D on Alternative Methods (CARDAM), Flemish Institute for Technological Research (VITO NV), 2400 Mol, Belgium; 7 Institució Catalana de Recerca i Estudis Avançats (ICREA), 08010 Barcelona, Spain

## Abstract

**Background:**

With the increasing use of nanomaterials, the need for methods and assays to examine their immunosafety is becoming urgent, in particular for nanomaterials that are deliberately administered to human subjects (as in the case of nanomedicines). To obtain reliable results, standardised *in vitro *immunotoxicological tests should be used to determine the effects of engineered nanoparticles on human immune responses. However, before assays can be standardised, it is important that suitable methods are established and validated.

**Results:**

In a collaborative work between European laboratories, existing immunological and toxicological *in vitro *assays were tested and compared for their suitability to test effects of nanoparticles on immune responses. The prototypical nanoparticles used were metal (oxide) particles, either custom-generated by wet synthesis or commercially available as powders. Several problems and challenges were encountered during assay validation, ranging from particle agglomeration in biological media and optical interference with assay systems, to chemical immunotoxicity of solvents and contamination with endotoxin.

**Conclusion:**

The problems that were encountered in the immunological assay systems used in this study, such as chemical or endotoxin contamination and optical interference caused by the dense material, significantly affected the data obtained. These problems have to be solved to enable the development of reliable assays for the assessment of nano-immunosafety.

## Background

The potential benefits and the risks associated with the application of nanomaterials have been widely debated in recent years. The need to correctly assess nanoparticle (NP) risks in order to protect workers, consumers and the environment is well accepted in the scientific and regulatory community [1,2]. Both the human population and the environment may be exposed to nanomaterials during all stages of the NP life cycle: raw material production, transport and storage, industrial use, consumer use, and waste disposal. The consumer use can vary from products like coated textiles or paints, where the presence of nano-products is not clearly stated, to sunscreens, where the NP content is explicitly labelled. In addition, medical use of NPs for diagnostic purposes or as drug delivery backbone represents intentional exposure to significant NP doses. Currently, a variety of methodologies are being discussed and evaluated to perform a complete risk assessment of nanomaterials. There are a number of European legislations that have the objective of implementing laws regarding use of and exposure to nanomaterials [3,4] including the REACH programme [5]. However, a lack of information on exposure levels, *in vitro *and *in vivo *NP effects and the life cycle of these entities make implementation of standards extremely difficult.

Even though a wealth of publications addresses the delicate issue of toxicity of engineered NPs [1,6,7], the exact events that occur in the interaction between NPs and the immune system are still largely unknown, even though nanoparticle-induced alterations of the immune system can have important effects on human health [8]. Despite a worldwide effort, results are overall contradictory, in particular when (immuno-) toxicity of NPs *in vitro *or *in vivo *is concerned, and no clear-cut information can be provided to the policy-makers, the producers and workers, and the public at large. Results obtained in different laboratories can often not be compared because of a lack of disclosure of experimental details as well as a lack of standardisation of methods and reagents.

An important aspect is that nanoparticle characterisation should also be performed at the point of use, since ageing, storage conditions and contamination can modify their properties in important ways. Alterations in particle characteristics can also occur when nanomaterials get in contact with the human body or with biological entities in the environment. Biological molecules can modify the nanomaterials and cause dissolution, aggregation or, at the very least, coating. The result can be anything from free ions or chemicals released from nanomaterials to micrometer-sized aggregates. Coating, for example by polysaccharides or proteins, may render the materials less harmful but can also change their properties in unexpected ways [9,10]. Furthermore, association with biological molecules such as endotoxins, can strongly affect the immunological response towards the particles.

For the analysis of the effects of nanoparticles on the immune system, the use of *in vivo *models can help in identifying possible risks for human health [11]. The adsorption, distribution, metabolism and excretion (ADME) of these materials may play a major role in immunotoxicity and can only be studied *in vivo *in animal models [12,13]. However, how well animal models reflect the real-life situation in humans and how well the results from these animal models correspond to the events observed after exposure of human beings to NPs is still to be determined. The use of *in vitro *assays with human cells could provide relevant information, which can be missed when using animal cells, in particular on the mechanisms of NP-mediated interactions and effects. For example, studies addressing the activation of toll-like receptors (TLR) by nanomaterials [14,15] should consider the significant differences in TLR expression, regulation and function between man and mouse [16].

Many publications describe the direct toxicity of NPs on human cells *in vitro*, using a plethora of different methods that either analyse the number of dead or alive cells or the metabolic capacity of the cells. These methods, however, miss to report the many events that can precede cell death, and overlook the large variety of effects that can take place without causing cell death.

The immune system is the complex of innate and adaptive mechanisms responsible for body's integrity, which can sense external or internal danger signals and initiate an appropriate defence response against such danger. Studying the interaction of NPs with the immune system is of particular relevance in the case of NPs used for medical applications, since they are often injected into the blood stream and come in direct contact with a multitude of immune cells [17]. The innate immune system provides the first line of defence, typically triggering a protective inflammatory response within minutes. If this proves insufficient, the slower adaptive response is induced. Deranged immune responses lead to pathology, both in the case of insufficient activity and in the case of uncontrolled reactions.

Most assays that are currently used to analyse nano-immunotoxicity were originally designed for the analysis of the immunotoxicity of dissolved chemicals. In many instances [8,18] these assays are not suitable for the purpose of nano-immunotoxicological analysis, and standard assay protocols applied to chemical toxicity analysis often cannot be used. New assays have to be designed for the analysis of nano-immunotoxicity and nano-immunomodulation.

In this study, metal or metal oxide NPs were used, many of which are used as core materials in medical applications. Gold NPs are currently tested for their use in medicine as drug transporters and for diagnostic imaging, *i.e. *for cancer detection [19]. Cobalt oxide NPs are used in a wide range of applications, such as contrast agents in magnetic resonance imaging [20], and silver NPs are used as anti-bacterial agents in wound dressings [21]. The study is specifically directed at identifying the problems that nano-immunotoxicologists can encounter when assessing the potential toxic and modulatory effects of NPs for immune cells in culture, in order to set up relevant and reliable assays for nano-immunosafety. To this end, we will focus on human cells that contribute to the innate immune defence mechanisms. These include professional immune cells, such as monocytes/macrophages, and non-professional defence cells, such as epithelial cells of the respiratory and gastro-intestinal tract.

## Methods

### NP synthesis, characterisation and preparation

#### Synthesis and characterisation

The inorganic NPs used in this work (Au, Ag, AgO, CoO, CeO_2_, Fe_3_O_4_) were synthesized by wet chemistry methods. All synthesis runs were carried out starting with organo-metallic and metallic salt precursors either decomposed or reduced in the presence of stabilisers using reagents from Sigma-Aldrich, Inc. (St. Louis, MO). Glass material was sterilised and depyrogenated prior to use to reduce the levels of lipopolysaccharide (LPS) and other contaminants in the NP preparations. NP dispersions were fully characterised by means of different techniques: Transmission Electron Microscopy (TEM), Zeta Potential measurements (Z-Potential), Dynamic Light Scattering (DLS), X-Ray Diffraction (XRD) and UV-VIS spectrophotometry. Table 1 reports the characteristics of the NPs synthesized and used in this work.

**Table 1:** Characteristics of NPs as synthesized and their solvents

Nanoparticle Composition	Mean Diameter (nm)	Surface Coating	Concentration			Solvent
				
			NPs/ml	μg/ml	nM	
Gold (Au)	4	Citrate ions	5 × 10^13^	56.7	83.1	Na Citrate 0.25 mM; oxidised NaBH_4 _0.3 mM; pH 8
Gold (Au)	13	Citrate ions	10^12^	62.7	1.7	Na Citrate 2.2 mM; pH 7
Gold (Au)	20	Citrate ions	10^12^	100.0	1.7	Na Citrate 0.85 mM; pH 7
Silver (Ag)	30	Citrate ions	10^12^	107.8	1.7	Na Citrate 10 mM; pH 7
Silver Oxide (AgO)	10-60	OH ions	10^12^	107.8	1.7	Oxidized NaBH_4 _2.64 mM; pH 9
Iron Oxide (Fe_3_O_4_)	7	TMAOH	10^14^	67.0	166.1	Tetramethylammonium Hydroxide (TMAOH) 5 mM; pH 10
Cobalt Oxide (CoO)	7	TMAOH	10^14^	38.1	166.1	TMAOH 1 mM; pH 9
Cerium Oxide (CeO_2_)	7	TMAOH	5 × 10^13^	14.0	83.1	TMAOH 1 mM; pH 9
Silver (Ag)	80	Citrate ions	1.1 × 10^9^	3.1	N.D.	Na Citrate 20 μM
Gold (Au)	50-100	None	N.D.	200	N.D.	None
Cerium Oxide (CeO_2_)	15-30	None	N.D.	200	N.D.	None
Cobalt Oxide (CoO)	28	None	N.D.	200	N.D.	None
Fe_x_O_y_	20-50	None	N.D.	200	N.D.	None

The 4 nm (mean diameter) Au NPs were synthesized following the procedure described by Jana et al. [22], based on the fast injection of 2.64 ml ice-cold freshly prepared sodium borohydride 0.1 M (NaBH_4_, reducing agent) into 200 ml of aqueous solution containing 0.25 mM gold tetrachloroaureate trihydrate (HAuCl_4_) and 0.25 mM trisodium citrate (stabilising agent), while stirring at room temperature. Other sizes of Au NPs (13 and 20 nm) were obtained with a procedure based on Turkevich et al. [23], consisting of the fast injection of a solution HAuCl_4 _to a boiling solution containing trisodium citrate under vigorous stirring. Varying the Au: sodium citrate ratio results in monodispersed Au NPs with different mean diameters [24]. All uncoated Au NPs were loosely coated with the negatively charged citrate ions. Ag NPs synthesis was based on the same Turkevich's method. For the 30 nm Ag NPs, 5 ml of sodium citrate 0.1 M were injected to a boiling solution of 50 ml of silver nitrate (AgNO_3_) 1 mM and stirred vigorously for 5 min. The resulting solution was cooled down in another vial to avoid deposition of silver on the glass surface. Oxidation of Ag NPs was negligible during the time frame of the experiments. Citrate ions were the coating agent as in the case of Au NPs. Colloidal CoO NPs were obtained after the synthesis of Co metallic NPs in organic solvent and further phase transfer. First, Co NPs of 7 nm mean diameter were prepared following the method described by Puntes et al. [25], based on the thermal decomposition of cobalt carbonyl (Co_2_(CO)_8_) in o-dichlorobenzene in the presence of oleic acid and trioctylphosphine, under controlled Ar-atmosphere. Thereafter, 1 mM tetra-methyl ammonium hydroxide (TMAOH) was used to exchange the NPs surfactant and thus render the particles water-soluble [26]. Under these conditions (exposed to air and fluid) the Co NPs slowly evolve towards cobalt oxide.

The CeO_2 _particles were synthesized by the nonisothermal precipitation procedure based on Zhou et al. [27] and Chen et al. [28] with some modifications. Briefly, 50 ml of cerium (III) nitrate solution (Ce(NO_3_)_3_•6H_2_O) 0.02 M were set at 70°C with a stirring rate of 500 rpm followed by the addition of 25 ml TMAOH 1 M. As soon as the TMAOH was added, the formation of white precipitates was observed. This stage was prolonged for 5 min, to oxidise the Ce(III)-Ce(IV). Right afterwards, the solution was rapidly transferred into a water bath, in which the reaction was continued at 50°C for 20 h. Then, the resulting solution was centrifuged, washed and resuspended in 50 ml TMAOH 1 mM to stabilise the formed CeO_2 _NPs.

For Fe_3_O_4 _NPs, Massart's method was followed [29,30]. Amounts of 1 mmol iron (II) chloride (FeCl_2_) and 2 mmol iron (III) chloride (FeCl_3_) were dissolved in 10 ml deoxygenated water and then added drop wise to 10 ml of a solution of 1 M deoxygenated TMAOH. After 30 min of vigorous stirring under a N_2 _stream, the Fe_3_O_4 _precipitate was washed by soft magnetic decantation, re-dissolved in 0.5 M TMAOH and diluted 100 times to obtain the final stable colloidal solution.

For the characterisation of the particles, TEM images were acquired with a JEOL 1010 Electron Microscope operating at an accelerating voltage of 80 kV. Samples for TEM were prepared by drop casting on carbon coated cooper TEM grids. The grids were left to dry at room temperature. Observations were made on different parts of the grid and with different magnifications and more than 400 particles were computer-analysed and measured for the size distribution.

Z-Potential measurement (Z-Potential) and Dynamic Light Scattering (DLS) measurements were made with a Malvern ZetaSizer Nano ZS Instrument operating at a light source wavelength of 532 nm and a fixed scattering angle of 173° for detection. Aliquots of 0.8 ml of the colloidal NP solutions were placed into the specific cuvette and the software was arranged with the specific parameters of refractive index and absorption coefficient of NP material and solvent viscosity (data required to obtain the correct value for each NP type). Z-Potential (surface charge) measurements are a commonly used tool to determine the stability of a colloidal suspension of electro-statically stabilised NPs. On the other hand, DLS allows the determination of the hydrodynamic diameter of colloidal particles and conjugates, that is the diameter of the sphere with the same Brownian motion as the analysed particle.

X-Ray Diffraction (XRD) spectra were acquired with a PANalytical X'Pert diffractometer that uses a Cu and Co Kα radiation source. Samples for XRD consist of the dry NPs in powder form. For this purpose, destabilisation of the NPs mixing the colloid with a solvent of different polarity was followed by soft centrifugation after which NPs precipitated. The supernatant was discarded, and the pellet of NPs was dried to eliminate all the moisture.

UV-visible spectrophotometry (UV-VIS) spectra were acquired with a Shimadzu UV-2400 spectrophotometer. One ml of the NP solution was placed in a cuvette, and spectral analysis was performed in the 300 nm to 800 nm range. This technique is widely used for metallic NPs, such as gold and silver, which exhibit a characteristic absorbance maximum in the visible range (the Surface Plasmon Resonance, SPR) that changes depending on the size and surface alterations. However, all the materials used absorb in the visible or UV range, making these measurements appropriate in all cases.

In addition, several NPs were obtained from commercial sources. Ag NPs (80 nm) were obtained from BB International Ltd. (Cardiff, UK) as colloidal suspension of 1.1 × 10^9 ^NPs/ml (corresponding to 3.1 μg/ml) in water with 20 μM citrate. Magnetite (iron oxide) NPs (100 nm) were provided by Chemicell (Berlin, Germany) as sterile suspension in water with citrate, at the concentration of 50 mg/ml (1.8 × 10^15 ^NPs/ml). Several dry NP powders were purchased from Nanostructured & Amorphous Materials (NanoAmor), Inc., Houston, TX, USA. According to the manufacturer's descriptions, Au nano-powder consisted of black spherical particles with diameters ranging from 50-100 nm. The surface area was 3.3 m^2^/g and sample purity was stated to be more than 99.99%. The CeO_2 _nanopowder was synthesized via sol-gel processing and consists of spherical, pale yellow particles with a size range from 15-30 nm, a surface area of 30-50 m^2^/g and a density of 7.1 g/cm^3^; the purity was 99.9%. Passivated, black and spherical cobalt oxide (CoO) particles were synthesized via Plasma Chemical Vapour Deposition (CVD). These particles had a diameter of 28 nm, a surface area of 40-60 m^2^/g, and a density of 8.9 g/cm^3^; the purity was 99.8% with small amounts of Ni (0.08%) and Fe (0.01%). Iron(II, III)oxide powder (Fe_x_O_y_; NanoAmor) comprised of spherical, reddish brown particles with a diameter of 20-50 nm, a surface area of about 50 m^2^/g, a density of 5.2 g/cm^3 ^and a purity of more than 98%. The dry particles were resuspended in endotoxin-free phosphate buffered saline to obtain particle suspensions with a final concentration as indicated for the different experiments. For most experiments, a volume of 10 μl of the NP suspension was added to 100 μl cell culture medium, resulting in a 9.1% v/v NP suspension, unless stated otherwise. For all the NP suspensions obtained by adding solvents to dry powders, we invariably observed that the particles aggregated and agglomerated, resulting in NP-derived micrometer scale materials (data not shown). The list of all NPs used in this study is reported in Table 1.

#### Preparation of NP solvents for biocompatibility in culture conditions

Initial experiments showed that the solvents (NP-free aqueous solution in which NPs are dispersed) used to synthesise and stabilise the particles in solution sometimes had a toxic effect on the different human cell lines used in our studies, even when used at a final concentration of 9.1% v/v. In order to avoid the analysis of chemical toxicity caused by the solvents instead of nanotoxicity, the cytotoxicity of all solvents in the absence of NPs was tested prior to the analysis of NP-induced immuno-toxicity effects. For this purpose, two sets of solvents were prepared and used as controls in the biological tests. One set of solvents was identical to the recipe of synthesis except for the precursor reagents, while the other was a NP solution from which NPs were removed by high-speed centrifugation. As discussed elsewhere [18], the analysis of these two solvents did not show relevant differences. Only those solvents that did not show any cytotoxic effect on THP-1 and A549 cells, as detected using the commercially available CellTiterBlue test (Promega, Madison, WI), were used for further analysis.

### Cell lines, primary cells and culture methods

#### A549 cell line and transfected reporter cell lines

The adherent human lung epithelial cell line A549 (DSMZ, Braunschweig, Germany) was cultured in RPMI-1640 medium supplemented with L-glutamine (4 mM), penicillin and streptomycin (each at 100 μg/ml) and 10% (v/v) heat-inactivated foetal bovine serum (FBS) (all from PAA Laboratories, Pasching, Austria). Cells were maintained at 37°C in humid air with 5% CO_2_. Stably transfected A549 cell lines were established and cultured as previously described [31]. In this study, A549 cell lines containing the IL-6 or the IL-8 promoter sequence linked to luciferase were used.

#### BEAS-2B cell line

Adherent human bronchial epithelial BEAS-2B cells (ATCC, LGC Promochem, Teddington, UK) were originally isolated from normal bronchial epithelium and immortalised in culture with an Ad12SV40 hybrid. The cells were cultured in T-75 flasks pre-coated with a mixture of 0.01 mg/ml human plasma fibronectin (Invitrogen, Paisley, UK), 0.03 mg/ml PureCol™ (Inamed Biomaterials, Fremont, USA) and 0.01 mg/ml bovine serum albumin (Sigma-Aldrich) dissolved in complete growth medium. The latter consisted of bronchial epithelial cell basal medium (BEBM; Lonza, Basel, Switzerland) supplemented with the BulletKit obtained from Lonza. Cells were grown in a humidified atmosphere at 37°C and 5% CO_2_. Before reaching 80% confluence, cells were sub-cultured using 0.25% (v/v) trypsin/0.53 mM versene solution (LGC Promochem) containing 5 mg/ml polyvinyl-pyrrolidone (Sigma-Aldrich).

#### THP-1 cell line

THP-1 (ATCC) is a human acute monocytic leukaemia cell line that lacks membrane bound immunoglobulins. THP-1 cells were cultured in RPMI-1640 medium supplemented with 2 mM L-glutamine, penicillin and streptomycin (1 IU/ml each), 10 mM HEPES and 10% heat-inactivated FBS (all from PAA Laboratories) and maintained at 37°C in humid atmosphere with 5% CO_2_.

#### CaCo-2 cell line

The adherent human gut mucosal epithelial cell line CaCo-2 (human colon carcinoma, Cell Bank ICLC-IST, Genoa, Italy) was cultured in DMEM medium supplemented with 2 mM L-glutamine, 50 μg/ml gentamicin (all from GIBCO, Invitrogen), and 10% heat-inactivated FBS (HyClone, Utah, USA). Cells were maintained at 37°C in humid air with 5% CO_2_. Before reaching 80%-90% confluence, cells were sub-cultured using 0.25% (v/v) trypsin. Medium was refreshed every 4 days. CaCo-2 cells represent human enterocytes after spontaneous *in vitro *differentiation. Upon reaching confluence, the proliferation rate gradually slowed until stopping, and cells formed an organised epithelial layer with the morphological and functional characteristics of a differentiated epithelium [32]. Therefore, cells were differentiated by maintaining them in culture for 15 days without passage or manipulation, and used afterwards for experimental purposes in culture medium with 5% (v/v) heat-inactivated human AB serum (Sigma-Aldrich).

#### Human primary monocytes

Human CD14^+ ^monocytes were isolated from peripheral blood mononuclear cells (PBMCs) obtained from buffy coats of healthy donors (Transfusion Centre, Cisanello Hospital, Pisa, Italy) by magnetic cell separation using the MIDIMACS technique according to the manufacturer's instructions (Miltenyi Biotec, Bergisch-Gladbach, Germany). The monocyte purity (>98%) was assessed by flow cytometry (FACScan; Becton Dickinson, Rutherford, NJ) and validated by microscope observation of like-stained slides stained with a modification of the May-Grünwald-Giemsa staining (Diff Quik; Medion Diagnostics, Düdingen, Switzerland). Cells were cultured in RPMI-1640 - Glutamax-I medium (GIBCO, Invitrogen) supplemented with 50 μg/ml gentamicin and 5% heat-inactivated AB human serum at 37°C in humid air with 5% CO_2_.

### Cell viability, cytotoxicity and cell proliferation assays

#### Metabolic activity - WST-1 assay

The mitochondrial function of cells exposed to NPs was analysed using the WST-1 assay (Roche Diagnostics, Basel, Switzerland). A549 cells were seeded in a 96-well plate at 1 × 10^4 ^cells/well/0.1 ml and incubated overnight. Ag NPs (80 nm; 3.09 μg/ml) were divided in two fractions. One fraction was centrifuged for 15 min at 10,000 rpm, and the pelleted NPs were resuspended in cell culture medium. Another fraction was used on cells directly, without solvent exchange. The solvent recovered after centrifugation was also used as control. To reach the final concentrations (ranging from 155 to 927 ng/ml), the NP suspensions were added to cell culture medium at a volume of 5-30 μl to reach a final volume of 0.1 ml. After 24 h of exposure to NPs, the WST assay was performed as described by the distributor. The net absorbance change at 450 nm taken from the wells of untreated cells was taken as 100% cell viability.

#### Cell proliferation - BrdU assay

The proliferation rate of the cells exposed to NPs was analysed using the BrdU colorimetric assay kit (Roche Diagnostics). A549 cells were seeded in 96 well plates at 1 × 10^4 ^cells/well/0.1 ml medium followed by overnight incubation. Cells were exposed to Ag NPs either suspended in water or re-suspended in culture medium at concentrations ranging from 155 to 927 ng/ml, by adding 5-30 μl of NP suspensions to the wells to reach a final volume of 0.1 ml. After 24 h of exposure, the BrdU assay was performed according to the manufacturer's instructions and analysed at 450 nm. Each experiment included, as control, cells lysed with 1% Triton X-100.

#### CellTiter-Blue (CTB) cell viability assay

To determine the effects of nanoparticles on cell viability the CTB assay from Promega was used. 100 μl of cells were plated out in 96-well microtiter plates (Corning Incorporated, Corning, NY) at a density of 5 × 10^3 ^cells/ml for A549 cells, or 5 × 10^4 ^cells/ml for THP-1 cells. A549 cells were plated out one day in advance in flat 96-well plates and left overnight to adhere and obtain their normal morphology. THP-1 cells were plated out on the same day as the particles were added in round bottom 96-well plates. Thereafter, 10 μl of the different NP suspensions at scalar concentrations were applied to the cells (9.1% v/v, at final mass concentrations of 200, 40, 8 and 1.6 μg/ml). The cells were then incubated for 48 h at 37°C in 5% CO_2_. Thereafter, the CTB-assay was performed according to the manufacturer's protocol and the fluorescence signal of resorufin was detected at 590 nm using a plate-reader (Tecan, Salzburg, Austria).

#### Toxilight cytotoxicity assay

The Toxilight assay (Lonza, Cologne, Germany) is a bioluminescence-based cytotoxicity assay that quantitatively measures the release of adenylate kinase (AK) from damaged cells. The cells were cultured as described for the CTB assay. After the incubation period, untreated cells (no particles) of 3 wells were lysed with 10 μl 10% Triton-X100 in water (Millipore, Billerica, MA) as a positive control. After 5 minutes incubation, the plates were centrifuged for 5 min at 400 × g. Thereafter the Toxilight assay was performed according to the manufacturer's instructions and luminescence was detected using a plate reader (Tecan).

#### Cytotox96 non-radioactive cytotoxicity assay

The Cytotox96 assay from Promega is a colorimetric-based cytotoxicity assay that quantitatively measures the release of lactate dehydrogenase (LDH) from damaged cells. The cells were cultured as described for the CTB assay. After the incubation period, untreated cells of 3 wells were lysed with 10 μl of 10% Triton-X100 in water (Millipore) as a positive control. After 5 min incubation the plates were centrifuged for 5 min at 400 × g. The Cytotox96 assay was then performed as described by the manufacturer and measured at 490 nm.

#### Neutral red cell viability assay

For assessment of effects on cell viability, the neutral red cytotoxicity assay was performed. BEAS-2B cells were seeded in pre-coated 96-well plates by adding to each well 200 μl of a cell suspension at 1 × 10^5 ^cells/ml, and the cells were grown to sub-confluence for 48 h. Cells were then exposed to 200 μl of the NP solutions or the corresponding solvents at 9.1% (v/v) in complete growth medium for 24 h. A solution of 1 mM paraquat (PQ) in culture medium was used as positive control. At the end of the exposure period the neutral red cytotoxicity assay was performed based on the INVITTOX protocol n°64 (ECVAM DB-ALM, http://ecvam-dbalm.jrc.ec.europa.eu/). The data, analysed at 540 nm, were expressed as percentage cell viability compared to unexposed control cultures (100% viability).

#### Cell damage and genotoxicity - micronucleus assay

Heparinised blood was obtained from two individual healthy donors (age <40 years, non-smokers, no medication for at least 2 weeks before donation) by venipuncture in Li-heparin tubes (Monovette^®^, Sarstedt AG & Co., Nümbrecht, Germany) according to standard medical protocol and with formal consent of the donors. The blood was diluted in RPMI-1640 medium supplemented with 10% FBS, 1.5% phytohaemagglutinin and 1% penicillin-streptomycin, and incubated for 24 h in culture tubes (Falcon, Becton Dickinson Labware, Franklin Lakes, NJ). Blood cells were then exposed to NPs for an additional 48 h, by direct addition in the culture tubes. Wet NPs were used at dilutions 9.1% v/v, and 10-fold dilutions of the original synthesis product (see Table 1). Dry NPs were resuspended in PBS at 2.0 mg/ml and used in culture at 0.2, 2.0, 20, and 200 μg/ml. For each experiment, a negative control (cells incubated with medium), a positive control (Mitomycin C 0.5 μM), and the solvent controls (cells incubated with medium containing solvent used for NP synthesis) were included. Cytochalasin B (6 μg/ml) was added after 44 h of culture (20 h after NP addition), in order to block cytokinesis and obtain binucleated cells, fundamental for the MN evaluation. After 48 h of incubation with NPs (72 h from start), cells were treated with an hypotonic solution for 3 min and subsequently prefixed in acid solution (3:5 methanol:acetic acid), washed with methanol and finally fixed with acid solution (6:1 methanol:acetic acid). The suspension was applied on pre-chilled slides and air-dried. Staining was performed with a 2% Giemsa solution. Slides were coded and analysed using an optical microscope (final magnification 400x). To assess the genetic damage, 2000 binucleated cells for each experimental point were examined, randomly coded, following the scoring criteria adopted by the Human Micronucleus Project [33]. Among these, the number of binucleated micronucleated (BNMN) leukocytes containing one or more micronuclei was counted. Moreover, in order to assess the viability index, 500 cells (mononucleated, binucleated and polynucleated) were scored to calculate the cytokinesis-block proliferation index (CBPI) according to Surralles [34].

### Detection of inflammatory cell activation

#### Cytokine gene expression by human primary monocytes

Human monocytes were seeded in a 6-well plate in 2 ml medium at the density of 5 × 10^6 ^cells/well. NPs were diluted in solvent and added to culture medium at a volume of 200 μl to reach a final concentration starting from 4.55% by volume (higher concentrations could not be used because of endotoxin contamination). Control cells were exposed to the corresponding NP solvents at 9.1% v/v (*i.e.*, 200 μl added to 2 ml). Exposure to NPs was also performed in parallel in the presence of LPS (endotoxin, 50 EU/ml; from *E. coli *055:B5; Sigma-Aldrich). Controls in all experiments were medium alone (negative control), and LPS alone (50 EU/ml; positive control). After different times of exposure to NPs, cells were collected and analysed for mRNA expression of cytokine and receptor genes by real-time PCR. Evaluated genes included the inflammatory cytokines IL-1β, IL-18, and the IL-18 receptor IL-18Rα. Total RNA was extracted from 4-5 × 10^6 ^human monocytes using the MicroRNeasy Kit (Qiagen, Hilden, Germany). RNA integrity was evaluated with a Bioanalyzer 2100 (Agilent Technologies, Waldbronn, Germany). Only RNA samples with a RIN (RNA Integrity Number) value between 7 and 10 were used (highest quality intact RNA). Retrotranscription of 500 ng of total RNA was performed with the QuantiTect reverse Transcription Kit (Qiagen), according to manufacturer's instructions. For quantitative or semi-quantitative evaluation of expression, the retro-transcribed cDNA was amplified with a RotorGene 3000 real-time cycler (Corbett Research, Doncaster, Victoria, Australia) as follows: one initial step at 50°C for 2 min and 95°C for 15 min was followed by 40 cycles at 95°C for 15 sec, and 56°C for 30 sec, and 72°C for 30 sec. The reaction mix contained 2.5 μl cDNA, 300 μM of primers, water and Master Mix (QuantiTech Sybr Green PCR Kit, Qiagen) in a final volume of 25 μl. The primers for β-actin (housekeeping gene), IL-1β, IL-18, and IL-18Rα were designed on the sequences published in GenBank in two different exons, and synthesised by Eurofins MWG Synthesis GmbH (Ebersberg, Germany). Expression was calculated in two ways, with comparable results. Semi-quantitative evaluation of expression was calculated as: Efficiency target gene^Ct target gene^/Efficiency reference gene^Ct reference gene^. Where Efficiency is the amplification efficiency, the target gene is the gene under analysis, the reference gene is β-actin, and Ct is the threshold cycle. For a more quantitative calculation, a standard DNA curve was constructed and expression of each target gene (ng/reaction) was calculated as the ratio with expression of β-actin (ng/reaction).

#### Cytokine production by differentiated CaCo-2 cells

Differentiated CaCo-2 cells were seeded in a 6-well plate at the density of 5 × 10^5 ^cells/well in 2 ml medium. NPs suspended in solvent (see Table 1) were added to culture medium at a volume of 200 μl (dilution at 9.1%). Control cells were exposed to the corresponding NP solvents at 9.1% v/v (*i.e.*, 200 μl added to 2 ml). Cultures were prolonged for 15 days (low dose chronic exposure).

Controls in all experiments were medium alone (negative control), the inflammatory cytokine IL-1β (10 ng/ml; positive control), and commercial large Co NPs (originally dry powder of diameter 50-200 nm, depyrogenated and resuspended in endotoxin-free saline, which however were at least partially aggregated upon depyrogenation and addition to culture medium).

Cell culture supernatants were cleared of cellular debris and NPs by high speed centrifugation, and frozen at -80°C until assayed for cytokine production. The multiple analyses of angiogenesis and inflammatory secreted factors were performed by the Human Cytokine Antibody Array Panel A Kit and the Human Angiogenesis Antibody Array Kit (R&D Systems; Minneapolis, MN). The protein production was detected by measuring the ECL chemiluminescence intensity (Pierce, Rockford, IL) using a VersaDoc Imaging System (Bio-Rad Laboratories Inc, UK).

#### Luciferase assay using stably transfected A549 cell lines

The luciferase assay using different stably transfected A549 cell lines was performed as previously described [35]. Briefly, cells were plated in 96-well plates (100 μl/well) at a density of 5 × 10^3 ^per well and left overnight to adhere and reach their normal morphology. On day 2, the cells were exposed to rhTNF-α (20 ng/ml) or left untreated. Subsequently, 10 μl of the NP solutions were added and the cells were incubated for 48 hr. The luciferase assay was performed on cell lysates. Supernatant was removed from the wells and 50 μl passive lysis buffer (Promega) were added for 10 min according to the manufacturer's instructions. Then, 40 μl of the cell lysates were transferred to non-transparent white 96-well microtiter plates (Corning). Forty μl of luciferine (the luciferase substrate) were added to the cell lysate and the luminescence was determined immediately using a plate reader (Tecan).

### Statistical Analysis

Experiments were performed twice or more as indicated in the figure legends. Mean values ± the standard deviation (SD) were calculated and data sets were compared using the Student's *t*-test. A *p *value < 0.05 was considered as statistically significant.

## Results

### NP physical state in culture media

**Figure 1:**
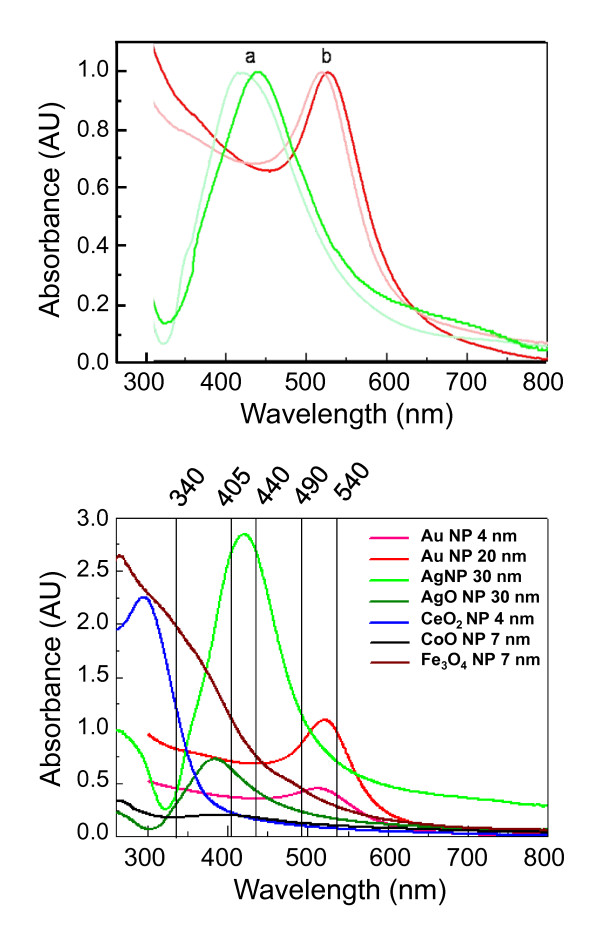
**Absorption spectra of NPs**. Upper panel: UV-visible spectra of Ag NPs (30 nm; a, red colour) and Au NPs (10 nm; b, green colour) exposed to cell culture medium (DMEM + 10% FBS) at 37°C. Fainter lines: NPs as synthesized. Darker lines: after 48 h in culture medium at 37°C. Red shift of the absorbance peak is an indication of protein corona formation. Lower panel: absorption spectra of the NPs used in this work. NP concentration is 1 × 10^12 ^NPs/ml for Au, Ag and AgO and 1 × 10^14 ^NPs/ml for CeO_2_, CoO and Fe_3_O_4_. At these concentrations, all NPs absorb in the visible range, where most of the biological tests have their readouts. In this figure: 340 nm is the absorption wavelength of NADH, measured in the LDH assay; 405 nm is the absorption wavelength of the chromophore p-nitroaniline (pNA) measured in the LAL assay for endotoxin determination; 440 nm, 490 nm and 540 nm are the readout for WST-1, MTS and MTT respectively.

NPs are readily coated with proteins when placed in cell culture medium. The evolution of the "protein corona" (PC) on the surface of some of the NPs used in this study has been described [36]. The formation of a PC will influence the cell responses, since cells initially interact with the particle surface, before coming in contact with the core material. Experimentally, the phenomenon can be observed as a red shift of the plasmon band, caused by the withdrawal of electron density from the surface of the particle due to the presence of the nucleophilic groups. An example is shown in Figure 1 (upper panel), showing the shift of UV-Vis absorption spectra of Au and Ag NPs upon protein coating.

**Figure 2:**
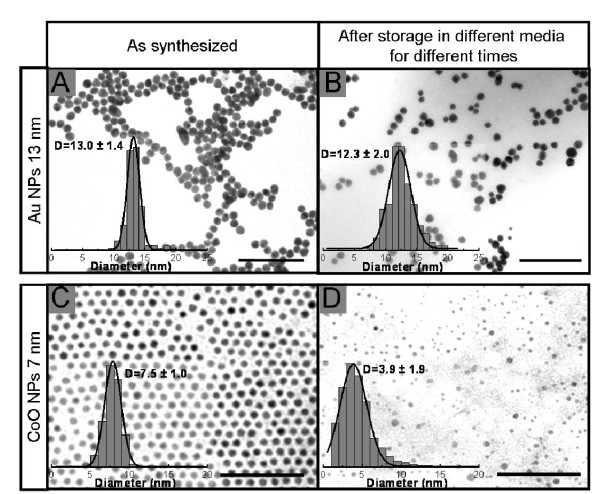
**TEM images and size distribution analysis of NPs exposed to different aqueous media**. Upper panels: 13 nm Au NPs: A, as synthesized; B, after 48 h incubation at 37°C with DMEM + 10% FBS. Lower panels: 7 nm CoO NPs: C, as synthesized in dichlorobenzene; D, after 24 h at room temperature after phase transfer to water. Scale bars are 100 nm.

NPs, as colloidal particles, are systems obtained from a chemical equilibrium, and exist in a meta-stable phase. Their final fate is the disintegration or agglomeration towards more stable phases [37]. Thus, even when the PC prevents the NPs from aggregation, the particles can still corrode. We have observed that all the inorganic NP preparations that were used in this work showed a release of cations with time (data not shown). Similar data were published for quantum dots (CdSe) [38] and carbon nanotubes [39], which corrode and release toxic Cd ions and less toxic carbon derivatives, respectively. Another consequence that should not be neglected, given the importance of size in the nano-bio interaction, is that corroded particles are much smaller in size compared to the original material. Striking evidence of this phenomenon is provided by the TEM images of Co NPs, showing that the morphology of the particles is severely affected 24 h after their dispersion in water (Figure 2). Spherical NPs with a homogeneous crystal contrast are transformed into shapeless NPs with a broader size distribution and polycrystalline nature. Together with the morphological transformation, an increase of Co ions in solution has been observed by inductively-coupled plasma mass spectrometry (up to 10% of the total NP mass; data not shown). This process is concomitant with the oxidation of Co towards CoO, a phenomenon that has been observed for all NPs. From the TEM images, a dissolution of 13% of the mass can be estimated for Co NPs incubated for 24 h at room temperature after phase transfer from just synthesized in dichlorobenzene to water, and of 11% of the mass for Au NPs after 48 h in cell culture medium (DMEM + 10% FBS) at 37°C.

### NP interference with the optically based assays' readout parameters

Many biological experimental readout parameters are based on transmission, *i.e.*, a detector measures the quantity of transmitted light at a given wavelength, and this measure is translated into an optical density. These systems have been designed for testing transparent matter, such as diluted cell lysates and cell culture media, and cannot be applied to optically dense materials such as NPs without accurate validation. Normally, NPs do not emit light but they can absorb it, thus resulting in optically dense samples. Density depends on their chemistry (*e.g.*, gold absorbs more light than iron oxide), size (larger NPs absorb more than small NPs) and concentration.

The NP density can increase the assay readout, leading to an over-estimation of the optical parameter. For example, in the case of a cell viability analysis that uses formazan, the optical density due to formazan (which is proportional to the number of living cells) can be significantly increased by the presence of NPs, giving the false impression of an improved viability and increased proliferation caused by the NPs [6,7]. In the case of toxic NPs, the decreased formation of formazan (due to reduced cell metabolism) could be masked by the NPs' optical density, providing a false impression of lack of toxicity. Small NPs (4-15 nm) of Au, Ag, AgO, Fe_3_O_4_, CeO_2_, and CoO, all absorb at the wavelengths used in most biological assay readouts: 340, 380, 405, 440, 540/550 nm (Figure 1, lower panel). In addition, some NPs can inhibit colour formation, thereby mimicking a cytotoxic effect. Indeed, it has been observed that carbon nanotubes can absorb formazan and protect it from metabolisation by the cells. The decreased colour formation, due to the direct effect of nanotubes on the dye rather than to decreased number of living cells, may thus lead to the false interpretation of a cytotoxic effect [40,41]. The best option, when possible, is to remove NPs before performing the assay (for instance when testing cell supernatants in ELISA assays). Although it has previously been shown that some nanomaterials, such as single walled carbon nanotubes, can indirectly interfere with such assays by adsorbing antibodies or cytokines [42,43], we have tested the adsorption of different cytokines on the metal and metaloxide particles used in this study and never found a reduction in the cytokine detection signal that could be attributed to cytokine binding to the particles. However, the appropriate controls, including cell free systems, have to be included to ensure the analysis of true NP-induced effects. In those cases for which this may not be possible (*e.g.*, in some viability assays), customised assays using wavelengths of 700-800 nm should be devised, or alternative assays that are not based on optical readouts should be used (*e.g.*, flow cytometry, visual counting of dead cells).

#### Toxicity assays

A major issue in devising nano-immunomodulatory assays is to make the NP suspensions compatible with the optimal conditions for cellular survival/growth in culture. Each type of culture medium and additive should be evaluated for possible interference with the endpoint measurements of assays aiming at evaluating cell death/proliferation/metabolic activity, as it is routinely done in assays for drug toxicity. However, additional care is needed in the case of nanotoxicity assessment, since NPs can significantly interact with medium components (*e.g.*, with serum proteins), and this interaction will vary depending on the medium composition and the NP characteristics.

In order to ensure that the effects found in any of the immunological tests were due to an immune-response and not to a loss of cell viability, the effects of NPs on the cell viability were determined using different commercial assays. The CTB assay is based on the ability of living cells to convert a redox-dye into a fluorescent product. The Toxilight assay is a bioluminescence-based cytotoxicity assay that quantitatively measures the release of adenylate kinase (AK) from damaged cells. The Cytotox96 assay is a colorimetric-based cytotoxicity assay that quantitatively measures the release of lactate dehydrogenase (LDH) from damaged cells. Using a combination of these three assays has the advantage that cell viability is determined at different levels, with different reagents and different readout systems.

**Figure 3:**
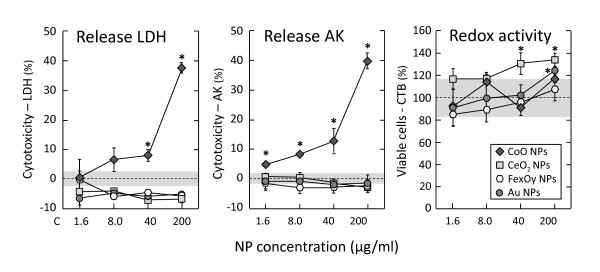
**Suitability of different cell viability assays for nanotoxicity testing**. Cytotoxicity of different NPs (suspended from dry powders) on THP-1 cells was tested after 48 h incubation using the Cytotox-96 assay from Promega (left panel) and the Toxilight assay from Lonza (center panel). In addition, the CellTiter-Blue assay from Promega was used to determine the cell viability (right panel). Data were normalised to the PBS control (9.1% v/v PBS) and mean values ± SD are shown. The dotted lines represent the values in control cells with PBS (taken as 0% cytotoxicity in left and centre panels, and as 100% viability in the right panel). The shaded areas represent the SD above and below the control values. * *p *< 0.05 *vs*. untreated control.

No significant cytotoxicity was found for any of the particles that were prepared in solution (see list in Table 1) when tested on A549 or THP-1 cells, independent of the assay used with a maximum incubation time of 48 h (data not shown). In addition to the particles that were synthesized in solution, a range of commercially available dry NPs were tested on different cell types. All types of dry particles formed aggregates after resuspension, resulting in larger particles in the μm range. These particles did affect the cell viability in some cases. As an example, data of cytotoxicity (LDH release or AK release) and cell viability (metabolic activity) of four different NP preparations are shown in the Figure 3 for the monocytic THP-1 cell line after 48 h incubation. Analysis of the LDH or AK release in the cell culture supernatant showed very clear cytotoxic effects of CoO NPs that were well reproducible and comparable in both assays (one based on colorimetric readings at 490 nm, and the other on bioluminescence). In contrast, the CTB assay was not suitable for this analysis. The fluorescence measurement showed an artificial increase of the cell viability, most likely due to the physical presence of the particles rather than to real particle-induced effects. The latter data are therefore considered to be an artefact and, since the viability of the cells is an important parameter in evaluating the extent of the immunomodulatory effects, they could lead to unreliable conclusions.

The optical interference was also observed in the Micronucleus (MN) assay used to assess genotoxicity of NPs on human PBMCs. NPs were added to the PBMCs either in monodispersed suspension (wet) or in powder form (dry). Experiments were carried out based on protocols originally designed to test chemical-induced genotoxicity, which were adapted to NP testing by considering some of the problems occurring (*e.g.*, aggregation and sedimentation) [44]. The use of this protocol showed that genotoxicity of wet NPs could be reliably detected and no obvious toxicity or genotoxicity due to the solvents or to the NP preparations was observed (Additional file 1, Figure S1). Super-imposable results were obtained with cells from different donors, underlining good assay reproducibility (data not shown).

For dry NPs, significant problems occurred during the sample preparation, with the formation of aggregates/agglomerates already occurring upon suspension in cell culture medium. After centrifugation and cell fixation for counting micronuclei in binucleated cells, large black spots were observed adhering to cells that practically covered them and did not allow micronuclei evaluation. The problem was not solved by sonication. At visual inspection, all samples contained large particles, irrespective of sonication, and the correlograms obtained by Dynamic Light Scattering analysis confirmed the high particle sedimentation of particles (data not shown). Therefore, strategies other than sonication are required to prevent NP aggregation/agglomeration.

### Immunotoxicity and immunomodulation induced by chemical or biological contaminants

Toxicity due to contaminants can interfere with the correct evaluation of the NP-induced effects. This might be due either to solvents or to endotoxin and other carry-over molecules. It is important to identify possible assay interference of contaminating molecules or, even worse, synergistic interactions between NPs and contaminants.

#### Solvent immunotoxic and immunomodulatory effects

The chemically synthesized NPs, as used in this study, are in a solvent that is normally not designed to be biocompatible. These solvents are intended to stabilise the particles in solution and avoid aggregation or agglomeration. Addition of solvent to the culture medium may induce direct cytotoxicity, or change the osmolarity and pH of the medium, thereby causing cell damage, and dilute nutrients, which decreases metabolic activity. Four citrate-stabilised monodispersed NP preparations (spherical Au NPs of diameter 4, 13, and 20 nm; and polydispersed Ag NPs of average size 30 nm; Table 1) were examined for their cytotoxic effect on human BEAS-2B lung cells using the neutral red assay. The four NP solutions contained different amounts of sodium citrate, giving final concentrations in the culture medium of 0.02 to 0.9 mM. Among the four NP preparations, only the one containing 0.9 mM sodium citrate (30 nm Ag NPs) showed significant toxicity, which overlapped the effect of the solvent alone, reflecting the effect induced by their respective solvents rather than the type, size and concentration of the NPs (data not shown).

**Figure 4:**
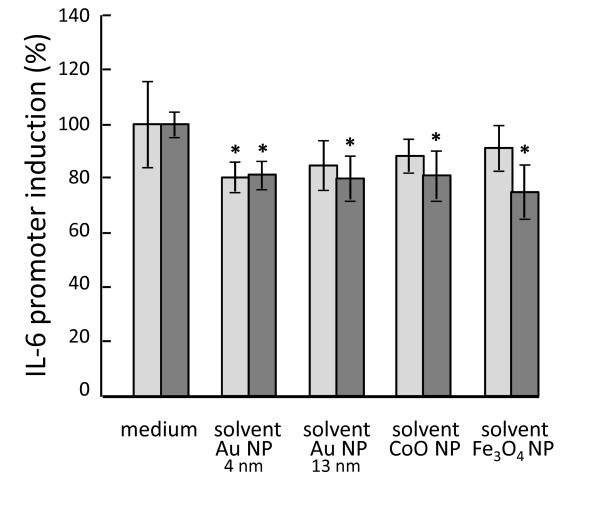
**Solvent immunotoxicity**. Four different particle solvents were tested for their immunomodulation on IL-6 promoter transfected A549 cells, either unstimulated or stimulated with TNF-α (20 ng/ml), after 48 h. For IL-6 promoter induction, the luminescence value of untreated cells was 220 ± 23 RLU (relative light units), while TNF-α-stimulated cells had a value of 1,485 ± 211 RLU. Data were normalised to allow a direct comparison between stimulated and unstimulated cells and to enable combining data from multiple experiments. * *p *< 0.05 *vs*. untreated control.

Another solvent-induced effect was observed with 80 nm Ag NPs (data not shown) on A549 cells. The different concentrations of NPs, resuspended at 1.1 × 10^9 ^NPs/ml (3.09 μg/ml) in water with 20 μM citrate, were obtained by adding increasing volumes of the NP suspension (5-30 μl) to the cells to reach a total volume of 100 μl (NP suspension + culture medium). Using the WST-1 assay, the number of metabolically active cells showed a dose-dependent tendency to decrease when exposed to the solvent alone, an effect that was not evident with the NP suspension. Replacing the solvent in the NP suspension with culture medium did not influence the lack of effect of NPs on A549 metabolic activity. Likewise, cell proliferation was apparently unaffected, or even increased by the Ag NPs suspended in solvent, while the solvent alone showed a tendency to be toxic at the highest concentration. This discrepancy between NP and solvent effects may be incorrectly interpreted as a protective effect of NPs, counteracting the toxicity caused by chemicals present in the solvent. As such, these experiments are an example of the risks of misinterpretations that could be incurred when overlooking key details. The toxicity is in this case likely due to the significant decrease in culture medium osmolarity caused by the addition of up to 30% v/v water, with consequent cell swelling and damage, while the "protective" effect of NPs most likely due to their optical density artificially masking the toxic effect of solvent. Based on these experiences, the solvent concentration was kept constant for all assays, independent of the NP concentration, to avoid the analysis of solvent effects instead of NP-induced effects. Using the different cell lines and the newly prepared biocompatible solvents, no significant cytotoxic effects could be detected when using 9.1% v/v of solvent. The effects of the different solvents on the immunological activation parameters were also analysed. All solvents included in this study (see Table 1) were tested on several different stable transfected A549 cells containing the sequences of cytokine promoters or the NF-kB binding sequence linked to the luciferase reporter gene. When these A549 reporter cells were incubated with the different solvents some small but significant effects on the induction of different cytokine promoters were observed [35]. As an example, the constitutive and TNF-α-dependent induction of the IL-6 promoter in A549 cells was shown to be inhibited by 20% for some solvents (Figure 4). Toxicity tests performed with the same cells and solvents indicated that this effect was not due to a loss in cell viability. These results indicate that the particle solvents can have immunomodulatory effects, in the absence of direct cytotoxicity.

**Figure 5:**
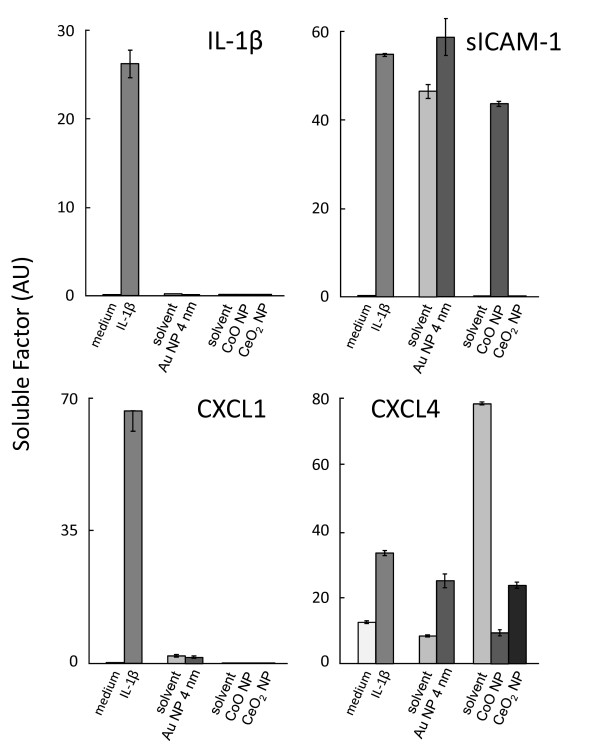
**Production of inflammation-related soluble factors by CaCo-2 cells**. Cells were chronically exposed for 15 days to culture medium alone or containing IL-1β (10 ng/ml, positive control), Au NPs 4 nm (5.2 μg/ml), CoO NPs (3.5 μg/ml), CeO_2 _NPs (1.3 μg/ml) (all corresponding to 9.1% v/v), or their solvents. Factors were detected in the cell supernatants by Proteome Profiler Antibody Array and evaluated as arbitrary units by densitometric analysis. Results for IL-1β, sICAM-1, CXCL1 and CXCL4 are reported in the figure, and presented as mean values ± SD from 2-4 replicate determinations.

Another example of solvent-induced immunomodulating effects is shown in experiments of chronic exposure of the human differentiated CaCo-2 gut mucosal cells to Au, CoO and CeO_2 _NPs. Cells were chronically exposed to NPs (6-7 μg/ml for Au 4 nm and CoO NPs, 0.15 μg/ml for CeO_2 _NPs, corresponding to a 9.1% dilution) or to their solvents (9.1% dilution) for 15 days, then supernatants were collected, centrifuged to eliminate residual NPs, and tested for the presence of inflammation-related soluble factors with semi-quantitative dot-blot-like assays (Proteome Profiler Antibody Arrays). Of the 84 factors tested, 40 were significantly produced by CaCo-2 cells or induced by stimulation with IL-1β, the positive control. The results for four of these factors are shown in Figure 5. Stimulation with IL-1β could induce production of three inflammation-related factors, IL-1β, sICAM-1 and CXCL1/GROα, and could increase the basal production of another factor, the chemokine CXCL4/PF4. In the case of IL-1β and CXCL1 production, chronic exposure to NPs or to their solvents did not have any effect (Figure 5, left panels). However, the solvent of Au NPs appeared to have a significant effect on sICAM-1 production, as potent as that of IL-1β itself (Figure 5, upper right panel). In this situation, the high increase of sICAM-1 production caused by the Au NPs cannot be considered as a true effect, as it is likely due to the solvent. On the other hand, the solvent of CoO NPs and CeO_2 _NPs (the same solvent for both NPs, see Table 1) had no effect on sICAM-1 production, thus the increase observed with CoO NPs (but not with CeO_2 _NPs) can be considered as a *bona fide *effect of the particles. Very different is the situation with the production of chemokine CXCL4 (Figure 5, lower right panel), as here the Au NP solvent is inactive, while the CoO/CeO_2 _NP solvent is highly effective. In this case, the increase caused by Au NPs can be considered true, whereas data obtained with CoO NPs and CeO_2 _NPs cannot be interpreted. The conclusion is that solvents can have unexpected effects in modulating cell activation, even when not directly toxic for cells, and that such effects are different depending on the biological endpoints measured. Thus, solvents must always be tested in parallel with NP preparations, even when they are devoid of direct toxicity.

Finally, it should be kept in mind that even when the solvents do not affect the viability of the cells and do not induce/modulate an immune response, there is still the possibility that the solvent and the NPs act in a synergistic manner, inducing in combination a higher or different effect compared to those found when analysing the single compounds.

#### Immunomodulating effects of endotoxin in NP preparations

**Figure 6:**
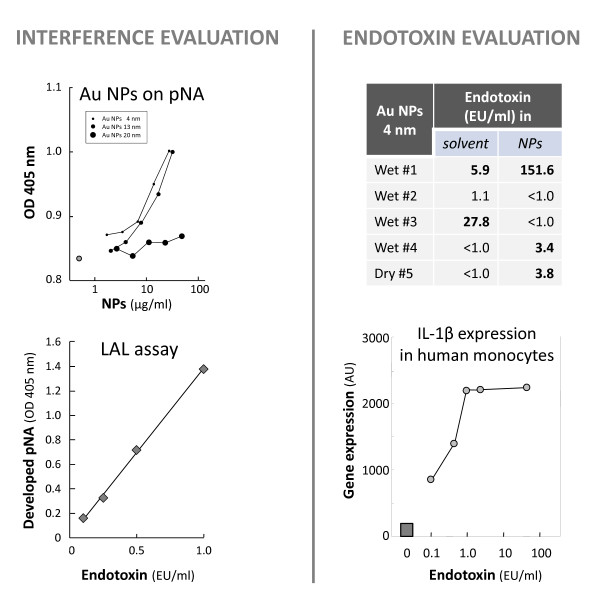
**Measuring endotoxin contamination in NP preparations**. As preliminary step, evaluation of the interference of Au NPs on the pNA readings at 405 nm was performed. Different concentrations of Au NPs (4, 13, and 20 nm diameter) could significantly increase the readout at OD_405 _of 125 μM pNA (round symbols) (*p *< 0.05 for all concentrations of NPs 4 nm, for the three highest of NPs 13 nm, and for the highest of NPs 20 nm) (upper left panel). The selected pNA concentration corresponds to that developed by 0.6 EU of endotoxin in the Endpoint Chromogenic LAL assay (lower left panel). The increase caused by NPs could therefore be misinterpreted as a significant increase in the presence of endotoxin. For this reason, endotoxin evaluation was then performed only on NP dilutions that did not cause significant interference with the pNA readings (typically, ≤ 1 μg/ml for Au NPs 4 nm, ≤ 4 μg/ml for Au NPs 13 nm, and ≤ 12.5 μg/ml for Au NPs 20 nm). Five separate batches of Au NPs 4 nm and their solvents were tested. For dry NPs, the solvent was endotoxin-free PBS. Batch-to-batch variability in the endotoxin contamination was evident (upper right panel). The importance of avoiding such contamination is shown by the powerful effect of minute amounts of endotoxin in activating IL-1β gene expression in human monocytes (lower right) (*p *< 0.05 for all endotoxin concentrations *vs*. control; square symbols).

Identifying the presence of biological contaminants in the NP preparations is important for the correct interpretation of the nano-immunotoxicological results [8]. Endotoxin contamination is the most common type of biological contamination in *in vitro *assays, even when working under sterile conditions, and can result in inflammatory responses. Testing for the presence of endotoxin is a common routine in biological laboratories, and many commercial assays are available. However, these assays should be validated for use with NPs, since most of them are based on optical readings and can be affected by the optical density of the NP samples [45]. Thus, before assessing endotoxin contamination, the suitability of the endotoxin assay must be checked. An example is given in Figure 6. Three preparations of Au NPs of different sizes (see Table 1 for characteristics) were tested for their interference with the optical detection at 405 nm of para-nitroaniline (pNA), the dye used as indicator of endotoxin presence in one of the most common assays (QCL-1000^® ^Endpoint Chromogenic LAL assay; Lonza). In this assay, increasing concentrations of standard endotoxin are detected as increased release of pNA, which is measured as OD increase at 405 nm, with a linear detection range between 0.1 EU/ml and 1.0 EU/ml (Figure 6, lower left panel). A concentration of pNA was selected (125 μM) corresponding to that developed in the LAL assay by 0.6 EU/ml of endotoxin. Au NPs were added to pNA and their OD_405 _was measured (Figure 6, upper left panel). It is evident that 4 nm and 13 nm Au NPs increase significantly the OD readings of pNA. The NP solvents were all optically inactive (data not shown). Thus, addition of 50% 4 nm Au NPs to pNA 125 μM increases the OD_405 _to a value that, in the LAL assay, would correspond to 0.73 EU/ml, as opposed to the initial 0.6 EU/ml. For Au NPs of 4 or 13 nm, these could never be used at dilutions less than ten-fold, without causing significant interference in the assay. With these cautions, by testing several batches of Au NPs 4 nm, it is evident that the presence of endotoxin is variable (possibly depending on the handling conditions during synthesis or to contaminated glassware), and in some cases the NPs or their solvent were heavily contaminated (Figure 6, upper right panel). To understand the risk of data misinterpretation that can be caused by an endotoxin contamination if going undetected, the activation of human monocytes by endotoxin is shown in terms of expression of the inflammatory cytokine IL-1β (Figure 6, lower right panel). It is evident that as little as 0.1 EU/ml of endotoxin can already induce significant gene expression, which becomes maximal between 1 and 50 EU/ml. Thus, as far as human monocytes are concerned, NP preparations can be tested only at dilutions that contain <0.1 EU/ml. It should be said that different cell types are differently sensitive to endotoxin, for instance human epithelial cells of gut and lung express very low levels of the endotoxin receptor TLR4 and are therefore relatively resistant to endotoxin effects, and thus less sensitive to endotoxin contamination in NP preparations (data not shown).

#### Synergy of contaminants with NPs

In addition to masking the effects/lack of effects of NPs, the presence of contaminants can synergise with NPs and induce unexpected cellular responses in immune cells. Taking again endotoxin as an example, in the case of human monocytes the type and kinetics of activation can be different in cells exposed to NPs in the presence of endotoxin, as compared to either stimulus alone. The kinetics of expression of two inflammatory genes, the cytokine IL-18 and its receptor IL-18Rα, have been evaluated in human primary monocytes exposed for 4 or 24 h to endotoxin-free AgO NPs (5 μg/ml, *i.e.*, 4.55% v/v), to endotoxin (50 EU/ml) or to both agents together (Table 2). Regarding expression of the IL-18 gene, this is rapidly induced by endotoxin at 4 h and down-regulated at later times (24 h). AgO NPs do not have a direct effect, nor can they affect the extent and kinetics of the endotoxin effect. When examining expression of another inflammation-related gene (IL-18Rα), again AgO NPs do not show any direct induction capacity, while endotoxin can induce gene expression only at later time points. However, when monocytes are exposed to particles and endotoxin together there is low but significant IL-18Rα gene expression already at 4 h, while the endotoxin-induced expression at 24 h is significantly reduced by the presence of particles. Thus, AgO NPs have no inflammatory effect by themselves on human monocytes, but their presence can modulate some aspects of the inflammatory defence response of monocytes to endotoxin. In real life the co-exposure of our immune system to more than one agent concomitantly is the rule, and the study of synergy/co-stimulation/antagonism is a highly relevant line of research in the field of nano-immunotoxicology.

**Table 2:** Synergy between NPs and endotoxin in causing biological effects in human primary monocytes

		**Gene expression (AU)**^**b**^			
**Treatment**^**a**^		IL-18		IL-18Rα	
			
		*no NPs*	*AgO NPs*	*no NPs*	*AgO NPs*
4 h	medium	0.77 ± 0.40	0.98 ± 0.20 ^n.s.^	0.49 ± 0.04	0.47 ± 0.13 ^n.s.^
	endotoxin	3.57 ± 0.80	3.04 ± 0.03 ^n.s.^	0.65 ± 0.16	1.78 ± 0.37*
24 h	medium	0.92 ± 0.11	0.92 ± 0.25 ^n.s.^	0.27 ± 0.12	0.82 ± 0.56 ^n.s.^
	endotoxin	0.31 ± 0.05	0.17 ± 0.05 ^n.s.^	18.56 ± 1.86	10.17 ± 2.01*

^a ^Human monocytes were exposed for 4 and 24 h to culture medium alone or to medium containing 50 EU/ml endotoxin. At the beginning of the incubation, cells were exposed to endotoxin-free AgO NPs (4.9 μg/ml, corresponding to a dilution at 4.55% v/v). Controls were cells exposed to culture medium alone or to a 4.55% dilution of the solvent (no detectable difference).

^b ^Gene expression was assessed by real-time PCR, using β-actin as housekeeping gene. Data are mean ± SD of replicate experiments.

* *p *< 0.05 *vs*. corresponding treatment in the absence of NPs; n.s. not significant *vs*. corresponding treatment in the absence of NPs

### Assays for detecting inflammatory effects: cytokine production and gene expression

As previously detailed, the presence of NPs (chemical composition, concentration, size and shape) can cause interference with the optical readouts of many assays for cellular functions. To assess the inflammatory effects of NPs, the production of soluble inflammatory cytokines and other factors is a well-established endpoint, which is routinely measured by ELISA. To avoid false positive or negative results caused by the physical presence of NPs in the cell supernatants, several actions can be taken. The first is that of centrifuging the cell supernatants before ELISA to sediment the NPs, a method that works well but that is time-consuming and involves a significant loss of sample volume. Another option is that of using molecular biology methods for detecting cell activation, *e.g. *measuring expression of inflammatory cytokine genes. Also in this case there might be problems related to the presence of NPs, such as a decreased efficiency of RNA extraction from NP-exposed cells, or a lower quality/integrity of extracted RNA. A solution, which however includes pre-determining which gene we want to look at, is the use of reporter cell lines in which a reporter gene (*e.g. *GFP or luciferase) is under the control of the promoter of the gene of interest. Luciferase is detected in the cell lysates using a standard luciferase assay, while GFP expression can be seen using a fluorescence microscope, and the presence of NPs can hardly interfere with the detection. Hereafter, we describe a series of problems and propose solutions for assessing cytokine expression/production in response to NPs.

#### RNA extraction and integrity

When measuring gene expression in NP-treated cells, a critical step is to assess the efficiency and quality of the RNA extraction procedure in the presence of NPs. The results in Table 3 show the recovery of RNA in samples of human monocytes exposed for 24 h to different types of NPs (all endotoxin-free and used at 4.55% v/v, corresponding to 0.7-4.9 μg/ml), a treatment that did not affect cell viability. It is clear that treatment with Au NPs 4 nm or with CoO NPs does not influence RNA recovery, which is practically identical to that achieved in untreated cells. The same holds true for Ag NPs, Au NPs (20 nm), and Fe_3_O_4 _NPs (tested only in one experiment; data not shown). As expected, RNA was efficiently recovered from cells exposed to the various NP solvents (Table 3). However, RNA extraction was clearly hampered in cells treated with CeO_2 _NPs, from which only about half of the RNA could be extracted. In cells exposed to AgO NPs, RNA recovery was highly variable, from completely normal to significantly impaired, in four different extractions. Despite the variability in RNA recovery, however, the integrity of the extracted RNA was always very high, indicating that the presence of NPs did not induce any particular damage or fragility to the nucleic acid (Table 3).

**Table 3:** RNA recovery and integrity from human monocytes exposed to NPs

**Treatment**^**a**^	**RNA recovery (% control ± SD)**^**b**^	**RNA integrity (RIN)**^**c**^
**Medium**	100.0	9.6
**Au NPs 4 nm**	96.3 ± 5.8	9.6
**AgO NPs**	78.8 ± 7.7	9.5
**CoO NPs**	97.2 ± 3.8	9.6
**CeO_2 _NPs**	56.5 ± 4.9 *	9.8
**Solvents (all)**	97.3 ± 3.4	9.5

^a ^Human monocytes (3-5 × 10^6 ^cells/well) were exposed for 24 h to culture medium alone or to 0.6-4.9 μg/ml (corresponding to 4.55% v/v) of different NPs or their solvents.

^b ^RNA recovery in control cells varied between 700 and 1400 ng/10^6 ^cells in seven separate experiments. Recovery in NP-treated cells is calculated as percent of the recovery in control cells (exposed to culture medium alone) within each single experiment, and expressed as mean ± SD. Recovery in cells treated with AgO NPs, tested in four experiments, was highly variable.

^c ^A value of RIN (RNA Integrity Number) between 7 and 10 indicates high quality intact RNA.

* *p *< 0.05 *vs*. medium control

#### Use of reporter cell lines

**Figure 7:**
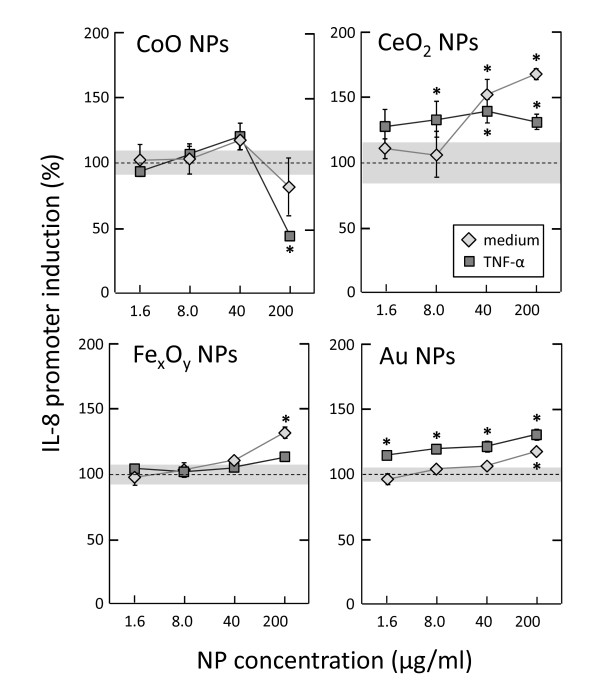
**NP-induced IL-8 promoter activation in A549 cells**. Four different NP powders were used to test their immunomodulatory effect on A549 cells, measured by the induction of the IL-8 promoter in transfected cells. Unstimulated cells (diamond shaped symbols) or 20 ng/ml TNF-α stimulated cells (square symbols) were exposed to increasing concentrations of NPs for 48 h. Mean values ± SD (n = 3) are shown. The dotted lines represent the values in control cells treated with PBS (100%). The shaded areas represent the SD above and below the control values. Untreated cells gave a luminescence value of 2,799 ± 367 RLU, while TNF-α-stimulated cells had a value of 100,366 ± 3,697 RLU. Data were normalised to the PBS control (9.1% v/v PBS; taken as 100% value) to allow a direct comparison between stimulated and unstimulated cells and to enable combining data from multiple experiments. * *p *< 0.05 *vs*. control

The A549 reporter cell lines containing the IL-6, IL-8 or TNF-α promoter sequence or 3 copies of the NF-κB binding sequence were used to analyse the inflammation-related effects of NPs. The effect of the wet particles on these reporter cell lines was low or absent as described in a previous publication [35]. The reporter cell lines proved useful in testing the immunotoxic and immunomodulatory effects of dry NPs, since aggregation and agglomeration of these particles did not influence the assay (the analysis is performed on cell lysates, and NPs can be eliminated by washing cells before lysis and centrifuging the lysates before analysis). The effects of four different dry NP preparations (Au, CoO, Fe_x_O_y _and CeO_2_) on the IL-8 promoter transfected A549 cell line are shown in Figure 7. Cells were either unstimulated or exposed to recombinant human TNF-α. CoO particles were found to decrease the IL-8 cytokine promoter induction, this reduction being most likely due to the decreased cell viability found when cells were incubated with CoO particles (data not shown). The other dry NPs tested did not affect the cell viability (Au, Fe_x_O_y _and CeO_2_, data not shown). Moreover, Au and Fe_x_O_y _particles did not have a major effect on the cytokine promoter induction. However, the CeO_2 _particles did significantly induce the IL-8 promoter. In unstimulated cells the induction was by 68%, while in cells activated with TNF-α a smaller increase was observed (31%). This is possibly due to the fact that TNF-α already induced a near-maximal activation (35-fold increase in the IL-8 promoter induction), thus largely masking the effect of the CeO_2 _NPs.

## Discussion

Nano-immunosafety is a key area of investigation for nanotoxicologists who aim at evaluating risks for the human health. Not only direct toxicity on immune cells, but even small alterations in the normal defence functions of our innate/inflammatory or adaptive immune responses could lead to a higher risk of developing diseases. Thus, simple, robust and representative assays are urgently required for assessing the possible impact of NPs on human immune functions. For this reason, the currently used *in vitro *assays and experimental tools need to be accurately optimised and validated, in order to adapt them for the analysis of NP-induced immune effects. This is not an obvious task, since both NPs and biological entities (*e.g.*, human immune cells) are complex systems highly reactive to changes in their environment. Since the physico-chemical characteristics of their respective environments are significantly different, modifications of the experimental conditions are necessary in order to bring the two systems together, and these may strongly alter the characteristics of the particles and the cells, a fact that commands for a rigorous validation of assay conditions.

A number of factors can affect the *in vitro *assays used for analysing the effects of NPs on immune cells and immune responses. A first source of variability, even before assays are performed, is represented by the great diversity of NPs used in different laboratories or even in one laboratory at different times. NPs can be synthesized by different methods, yielding products that can vary from batch to batch, contain variable amounts of catalysis residues, be more or less contaminated with biological contaminants, and with characteristics that can change due to the conditions and the time the particles are stored [46] (data not shown). Particle contamination can strongly affect the outcome of *in vitro *and *in vivo *experiments. As an example, toxicity of Diesel exhaust particles was significantly reduced when all chemical and biological contaminants were removed from their surface [47]. NPs used in the present study were suspended in different solvents, mainly containing sodium citrate or TMAOH. In agreement with previous studies [48], citrate showed a dose-dependent cytotoxic effect on BEAS-2B human primary lung cells. This kind of observation highlights the need for accurate studies addressing the possible confounding effects of chemical contaminants or solvent components on the assessment of true NP-related immune effects. Another type of contamination, of great importance when studying immunomodulating/immunotoxic effects of NPs, is the biological contamination. NP suspensions used in this study were sterile (*i.e.*, devoid of live bacterial contaminations), but nevertheless both NPs and their solvents contained variable levels of endotoxin (LPS, endogenous pyrogen; a component of the bacterial cell wall). Many immune cells (either cell lines or primary cells), in particular monocytes/macrophages, are very sensitive to endotoxin and can be readily activated by trace amounts of it. Endotoxin is a very common contaminant of glassware, culture media and additives (*e.g.*, FBS), which cannot be removed by sterilisation. While cell cultures are routinely controlled for endotoxin contamination, and handled exclusively with endotoxin-free equipment and reagents, NP synthesis is usually performed in chemical labs using non-decontaminated glassware and reagents. The way to effectively remove endotoxin is by incineration (for example by heating to 250°C for 30 minutes), but this treatment is not suitable for NPs as it induces irreversible agglomeration. This only leaves the option open of performing the entire synthesis process in endotoxin-free conditions and with the use of pyrogen-free materials [49]. Endotoxin levels should therefore be measured for each particle preparation, in order to ensure that the effects measured cannot be due to this highly active contaminant. Endotoxin is not the only bacterial compound able to induce inflammatory immune reactions, but its heat stability makes its presence more likely after sterilisation. The availability of several commercial assays makes identification of endotoxin easy, but close care should be taken that the particles do not interfere with the assays [45]. Moreover, the presence of significant amounts of endotoxin may also be an indicator of contamination with bacterial compounds in general, which may go undetected because of lack of suitable assays for their identification.

A major technical problem in the adaptation of immunoassays to the analysis of NP effects is the direct interference of NPs with the assay procedures and readouts. The optical density of NPs and their steric hindrance can interfere with the normal experimental procedures (*e.g.*, RNA extraction, any density gradient-dependent procedure) and, in particular, with the optically-based assay readouts [50]. As shown in the present study, to bypass the risk of misinterpretation of results it is recommended to use multiple assays in parallel and to include a number of controls (particles only, particles plus readout dye, analysis at time point zero, etc.), and/or to test NPs only at dilutions that do not cause interference.

In testing of NP-induced immune effects, the choice of the biological assay is also of central importance. Practical reasons, besides the general task of reducing animal experimentation, command the choice of *in vitro *assays. Given the complexity of immune responses, assays should be designed in such a way as to represent selected relevant real life situations. For instance, in the case of nanomedicines to be administered intravenously, representative *in vitro *cellular assays should be based on human blood leukocytes (such as monocytes), which are the first cells to come in contact with the injected NPs, and have biological endpoints representing the early innate/inflammatory type responses (see examples in Table 2). Likewise, in the case of ingested or inhaled material, the response of non-professional defence cells such as human gut and lung epithelial cells can be considered as representative of the real life situation (see for example Figure 5 and 7). Primary cells are the first choice in such assays, as they reproduce the response of normal cells of normal individuals. In most cases, the response of primary cells to stimulation is more sensitive than that of continuous cell lines and, in some instances, also qualitatively different [35]. Thus, whenever possible, primary cells are the best choice for setting up representative assays. This is the case for instance of human monocytes, which are easily accessible (peripheral blood) and which show responses to prototypical stimuli that are remarkably reproducible from donor to donor both qualitatively, quantitatively and kinetically (DB, data not shown). However, the use of primary cells is not always feasible (*e.g.*, in the case of primary lung epithelial cells) and is also hampered by the limited cellular life span, which requires obtaining fresh cells (most likely from different donors) for each assay. This makes standardisation very difficult, because of the difficulty in getting cells and the risk of donor-to-donor variability (except in special cases, such as blood monocytes). As an alternative, the use of cell lines (transformed or tumour cells with unrestrained proliferative capacity) is technically easier and reproducible, thus suitable to standardisation and applicable to a first-line screening of the immunomodulatory effects of NPs. Of course, an absolute requirement is that the selected endpoint (*e.g.*, activation of IL-8 expression) is representative of primary cell activation. In this respect, particular caution should be used in testing the anti-proliferative and cytotoxic effects of NPs. Indeed, transformed and tumour cells have a different cell cycle regulation and cell survival compared to primary cells, making them particularly unsuitable for cytotoxicity assays (apoptosis, necrosis, inhibition of proliferation, etc.). Ideally, every NP effect identified using cell lines should be validated on primary cells, unless the assay has already been validated.

## Conclusions

The studies reported here have focussed on *in vitro *immunoassays, in particular addressing early inflammatory/innate immune cellular responses, as those carried out by epithelial cells (barrier and active innate defence cells) and by monocytes/macrophages (professional innate/inflammatory defence cells). Information on the adsorption, distribution, metabolism and excretion (ADME) of nanomaterials is of main importance in understanding the potential toxicity and also in monitoring the biopharmaceutical effects in nanomedicine [12,13]. Studies have been performed with a multitude of different nanomaterials to determine the ADME in mice or rat models upon inhalation, injection or dermal application. These studies provide valuable information on the localisation of the nanomaterials in several different tissues, which underlies the distinctive pattern of effects. However, ADME studies cannot provide meaningful information on the features of interaction between nanomaterials and defence cells in tissues and organs, and on the mechanisms of the effects/immune reactions. In contrast, this information can be provided by *in vitro *experiments with isolated cells or single cell types. The use of single cells allows us to understand the role of each cell type in the observed immune responses, and will shed light on the mechanism behind the effects seen *in vivo*. Understanding the mechanisms is the starting point for a more focussed approach to optimise the safety of nanomedicines or to reduce the toxicity of nanomaterial-containing products. In addition, the *in vitro *mechanistic studies may serve as a basis for developing representative assays for assessing immunomodulatory effects of nanomaterials that correlate with the *in vivo *outcomes. Indeed, the rapid development of the nanotechnological industry has resulted in a huge expansion of the different nanomaterials available, which vary in chemical composition, shape, size, and surface coating. Safety testing for all these new materials with *in vivo *methods is both economically unfeasible and ethically unacceptable, as large numbers of animals would be required. Thus, standardised *in vitro *assays with selected cell types may be the best option as screening tools to identify potentially hazardous materials. At present, a comprehensive picture of nanomaterial-induced effects cannot be obtained unless both *in vivo *and *in vitro *tools are used.

## Competing interests

The authors declare that they have no competing interests.

## Authors' contributions

GJO was involved in the experimental design of the CTB, Toxilight, Cytotox-96 and promoter cell line based assays and was heavily involved in the preparation and the revision of the manuscript. EC prepared the NPs in solution, performed a main part of the characterisation and wrote the parts describing the NP synthesis and characteristics. PI performed the assays with the CaCo2 cells and was involved in the experiments with the human monocytes, RC was involved in the optimisation of the genotoxicity assays and in the preparation of the manuscript, RS performed the CTB, Toxilight, Cytotox-96 and promoter cell line based assays and was involved in writing of the manuscript, JP performed the genotoxicity assays and was involved in writing the manuscript, TP has made substantial contributions to the conception of the manuscript and performed preliminary experiments, YK performed the WST assays for the A549 cells and was involved in writing the manuscript, DO performed the BAES-2B experiments and critically revised the manuscript, FF performed the assays with the human monocytes and was involved in the experiments with the CaCo-2 cells, HL performed the BAES-2B experiments and critically revised the manuscript, DL performed the assays with the CaCo2 cells and was involved in the experiments with the human monocytes, FR participated in the coordination of the study and the design of the experiments, IN participated in the coordination of the study and the design of the experiments and was involved in writing the manuscript, HT participated in the coordination of the study and the design of the experiments and was involved in writing the manuscript, VFP participated in the coordination of the study and the design of the experiments and was involved in writing the manuscript, AD participated in the coordination of the study and the design of the experiments and critically reviewed the manuscript, DB participated in the coordination of the study and the design of the experiments and was heavily involved in the preparation and revision of the manuscript.

All authors read and approved of the final manuscript.

## Supplementary Material

Additional file 1 **Genotoxic effects of selected NPs on human peripheral blood leukocytes**. Cells in suspension were treated for 48 h with NPs or their respective solvents. Genotoxicity was evaluated as the number of binucleated micronucleated (BNMN) leukocytes every 1000 cells from two separate donors. Positive controls (treated with Mitomycin C 0.5 μM) contained >100 BNMN cells/1000 cells (not shown). Representative data reported in the figure refer to cells from one of the two donors treated with the highest NP/solvent concentration (9.1%). Final NP concentrations in the assay were the following: Au NPs (13 nm) 5.7 μg/ml; CoO NPs 3.5 μg/ml; Fe_3_O_4 _NPs 6.1 μg/ml; CeO_2 _NPs 1.3 μg/ml; Ag NPs 9.8 μg/ml. Click here for file
