# Supplementary figures and images for: Problems and challenges in the development and validation of human cell-based assays to determine nanoparticle-induced immunomodulatory effects
Source: Part Fibre Toxicol. 2011 Feb 9;8:8. doi: 10.1186/1743-8977-8-8 (PMC3045340; doi:10.1186/1743-8977-8-8)

## Slide 1
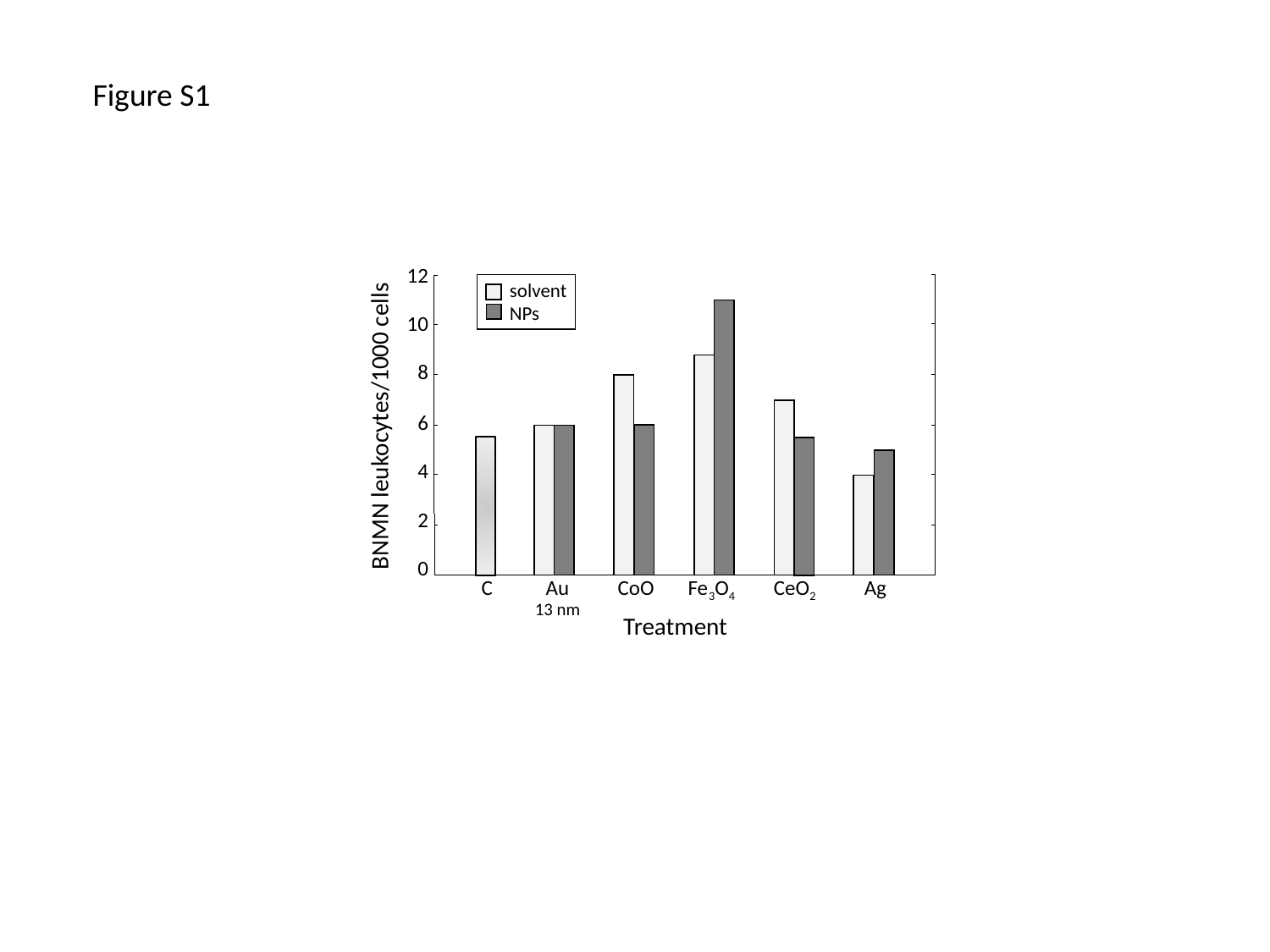

Figure S1
BNMN leukocytes/1000 cells
12
10
8
6
4
2
0
solvent
NPs
 C Au CoO Fe3O4 CeO2 Ag
 13 nm
Treatment

Supplement: Additional file 1 — Genotoxic effects of selected NPs on human peripheral blood leukocytes. Cells in suspension were treated for 48 h with NPs or their respective solvents. Genotoxicity was evaluated as the number of binucleated micronucleated (BNMN) leukocytes every 1000 cells from two separate donors. Positive controls (treated with Mitomycin C 0.5 μM) contained >100 BNMN cells/1000 cells (not shown). Representative data reported in the figure refer to cells from one of the two donors treated with the highest NP/solvent concentration (9.1%). Final NP concentrations in the assay were the following: Au NPs (13 nm) 5.7 μg/ml; CoO NPs 3.5 μg/ml; Fe3O4 NPs 6.1 μg/ml; CeO2 NPs 1.3 μg/ml; Ag NPs 9.8 μg/ml. [file 1743-8977-8-8-S1.PPT]
